# Workflow and imaging strategies for real-time MR-guided atrial transseptal puncture

**DOI:** 10.1093/ehjimp/qyaf117

**Published:** 2025-10-31

**Authors:** Luuk H G A Hopman, Michiel J B Kemme, Pranav Bhagirath, Raschel D van Luijk, Vjeran Karloci, Cornelis P Allaart, Marco J W Götte

**Affiliations:** Department of Cardiology, Amsterdam UMC, De Boelelaan 1118, Amsterdam 1081 HV, The Netherlands; Department of Cardiology, Amsterdam UMC, De Boelelaan 1118, Amsterdam 1081 HV, The Netherlands; Department of Cardiology, Amsterdam UMC, De Boelelaan 1118, Amsterdam 1081 HV, The Netherlands; Department of Radiology, Amsterdam UMC, Amsterdam, The Netherlands; Department of Interventional CMR, Imricor Medical Systems, Burnsville, MN, USA; Department of Cardiology, Amsterdam UMC, De Boelelaan 1118, Amsterdam 1081 HV, The Netherlands; Department of Cardiology, Amsterdam UMC, De Boelelaan 1118, Amsterdam 1081 HV, The Netherlands; Division of Cardiology, Department of Cardiac Sciences, Cumming School of Medicine, Libin Cardiovascular Institute, University of Calgary, Calgary, Canada

**Keywords:** interventional MRI, cardiac MRI, transseptal puncture, ablation

## Abstract

Interventional cardiac magnetic resonance imaging (iCMR) offers distinct advantages for guiding complex cardiac procedures, including 3D visualization, soft tissue characterization, and avoidance of ionizing radiation. Transseptal puncture (TSP), essential for left heart access, poses specific challenges under MR-guidance. The development of MR-compatible TSP sets comprising non-ferromagnetic sheaths, dilators, and needles, represents a major step toward safe execution of TSP in the MRI environment. This report provides practical, step-by-step guidance for MR-guided TSP, focusing on imaging strategies and integration of advanced 2D and 3D navigation tools. Real-time cine imaging in dedicated planes enables precise localization of the fossa ovalis, confirmation of septal tenting, and avoidance of adjacent structures. Complementary use of a vendor-neutral MR-compatible 3D navigation system allows dynamic catheter tracking within a segmented static 3D anatomical shell, enhancing spatial orientation and procedural accuracy. Feasibility was demonstrated in a porcine model, where an MR-compatible sheath and trackable dilator were successfully navigated to the interatrial septum and TSP was achieved, enabling left atrial (LA) access. Subsequent mapping confirmed catheter positioning within the LA. Remaining challenges include limited guidewire visibility, low image temporal resolution compared with fluoroscopy, and the investigational status of current MR-compatible TSP sets. These factors must be addressed before clinical translation. In conclusion, MR-guided TSP using dedicated imaging planes and MR-compatible devices is technically feasible and may facilitate future radiation-free left heart interventions. Continued device refinement, including improved passive instrument visibility and active tracking technologies, faster real-time cine imaging, and regulatory approval are critical for safe and widespread clinical adoption.

## Graphical Abstract

**Graphical Abstract:**
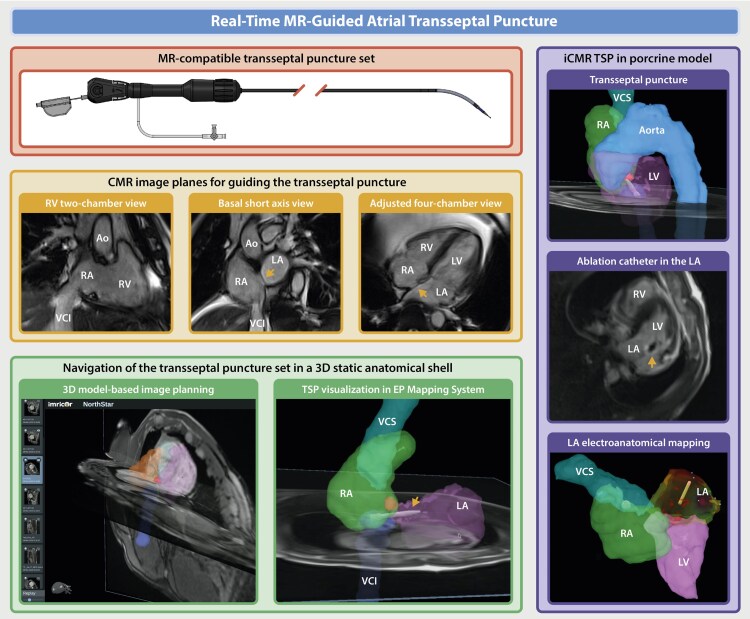

## Introduction

Interventional cardiac magnetic resonance imaging (iCMR) offers several advantages for guiding complex cardiac interventional procedures, including three-dimensional (3D) visualization, real-time characterization of soft tissue properties, and avoidance of ionizing radiation. These attributes position iCMR as an alternative strategy for improving outcomes in established interventional procedures and the development of novel cardiac interventions, particularly in arrhythmia ablation, pressure measurements in congenital heart disease, and targeted biopsies for infiltrative cardiomyopathies.^1^

**Figure 1:**
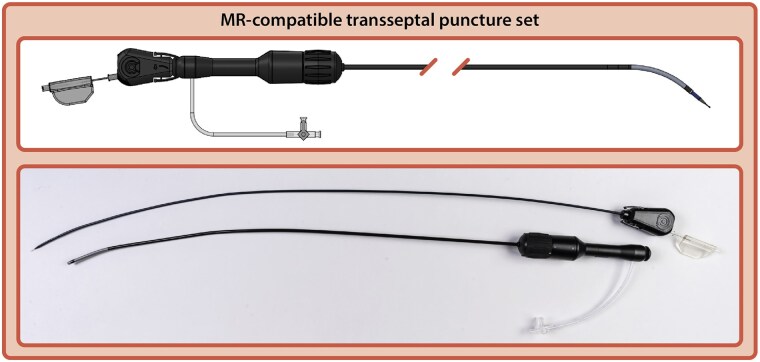
MR-compatible TSP set. Computer-aided design and picture of an MR-compatible TSP set (Imricor Medical Systems, Burnsville, MN, USA) consists of a dilator, steerable sheath and transseptal needle.

As the use of iCMR continues to expand, new procedural demands and technical challenges arise. Transcatheter needle puncture of the interatrial septum (transseptal puncture, TSP), a manoeuvre for accessing the left side of the heart, represents such a challenge.^2^ The development of MR-compatible TSP sets, with components already being tested in a research setting, is a significant advance in this regard.^3^ The MR-compatible TSP sets are designed specifically for use in an MRI environment, ensuring minimal magnetic interference and maintaining imaging quality. These sets, typically made of non-ferromagnetic materials such as titanium and polymers, include key components such as an MR-compatible sheath, dilator, and puncture needle (*Figure 1*).

Importantly, the introduction of MR-compatible TSP sets require dedicated imaging and procedural planning strategies tailored for safe and effective MR-guided TSP. In this short report, our primary objective is educational: to provide practical, step-by-step guidance for operators on how MR-guided TSP can be performed, supported by relevant imaging examples. While this work is not intended to present clinical outcome data, it aims to bridge the gap between technical feasibility demonstrated in a preclinical model and future clinical translation. Therefore, we provide an overview of relevant cardiac imaging planes, both in two-dimensional (2D) and 3D formats, and discuss their application in facilitating real-time MR-guided TSP using an MR-compatible TSP set.

## MR-guided TSP and navigation workflow

### Standard TSP workflow

The standard TSP procedure begins with venous access via the femoral vein, followed by introduction of a guidewire via the inferior vena cava (IVC) and right atrium (RA) into the superior vena cava (SVC). A sheath and dilator are then advanced over the guidewire into the RA.^2^ Although passive markers that create localized signal voids can make guidewires visible on MRI, reliably visualizing the guidewire remains a significant technical challenge. Nonetheless, it is critical for procedural safety, as advancing the sheath or dilator without wire support poses a substantial risk of vascular or tissue injury. Once the guidewire has been replaced with the MR-compatible transseptal needle, further advancement should only occur if the catheter tip is properly aligned against the interatrial septum, typically achieved by pulling the sheath and dilator back from the SVC into the fossa ovalis.

### MR imaging planes for safe TSP

**Figure 2:**
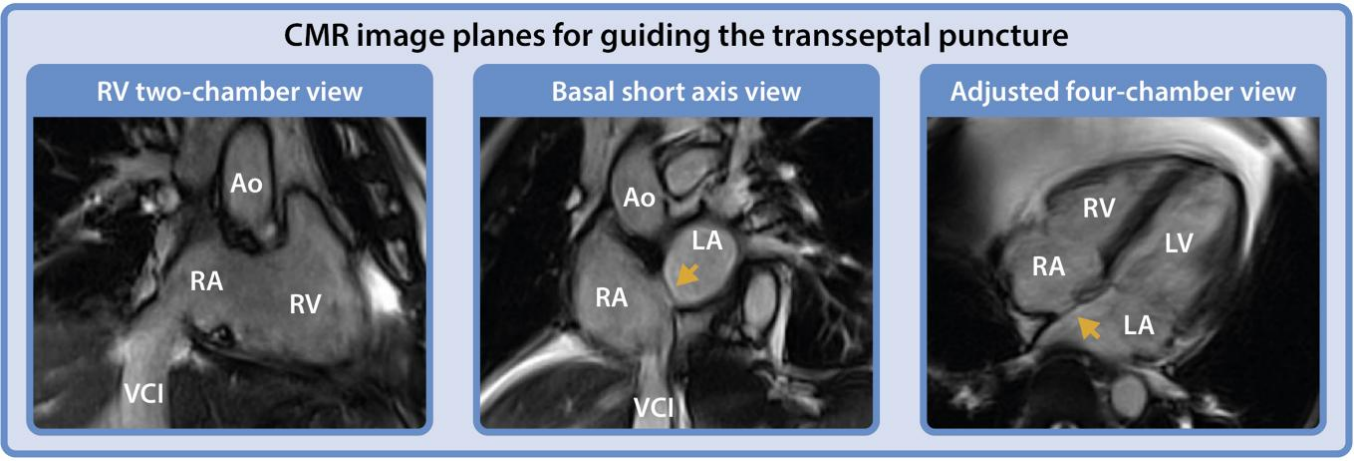
CMR image planes for guiding the TSP. RV two-chamber view (left panel) visualizing both the RV inflow and outflow tract (comparable fluoroscopy orientation: RAO); the basal short axis view (mid panel) (comparable fluoroscopy orientation: LAO), and four-chamber view (right panel). The arrow indicates the foramen ovale. Ao, Aorta; LA, left atrium; LV, left ventricle; RA, right atrium; RV, right ventricle; VCI, inferior vena cava; VCS, superior vena cava.

While in fluoroscopy-guided procedures, left anterior oblique (LAO) and right anterior oblique (RAO) views, or adjunctive imaging (e.g. transoesophageal echocardiography or intracardiac echocardiography) are used to confirm device position, MR-guided TSP relies exclusively on dedicated MR imaging planes. Key imaging planes essential for safe and effective MR-guided TSP are considered the adjusted right ventricular (RV) two-chamber view (comparable to RAO in fluoroscopy view), basal short axis view (comparable to LAO in fluoroscopy view), and four-chamber view. The RV two-chamber view facilitates navigation of the dilator within the RA, while the adjusted basal short axis view provides clear delineation of the interatrial septum, essential for visualizing septal tenting during the application of forward pressure.^4^ This imaging perspective is crucial to ensure the catheter remains correctly oriented toward the interatrial septum, avoiding inadvertent puncture of both the adjacent aortic root anteriorly or the posterior wall. Finally, the four-chamber view provides orthogonal confirmation of the dilator’s position at the septum, allowing precise alignment with the axial plane and enhancing procedural safety (*Figure 2*).

### Incorporation of 3D MR-compatible navigation systems

**Figure 3:**
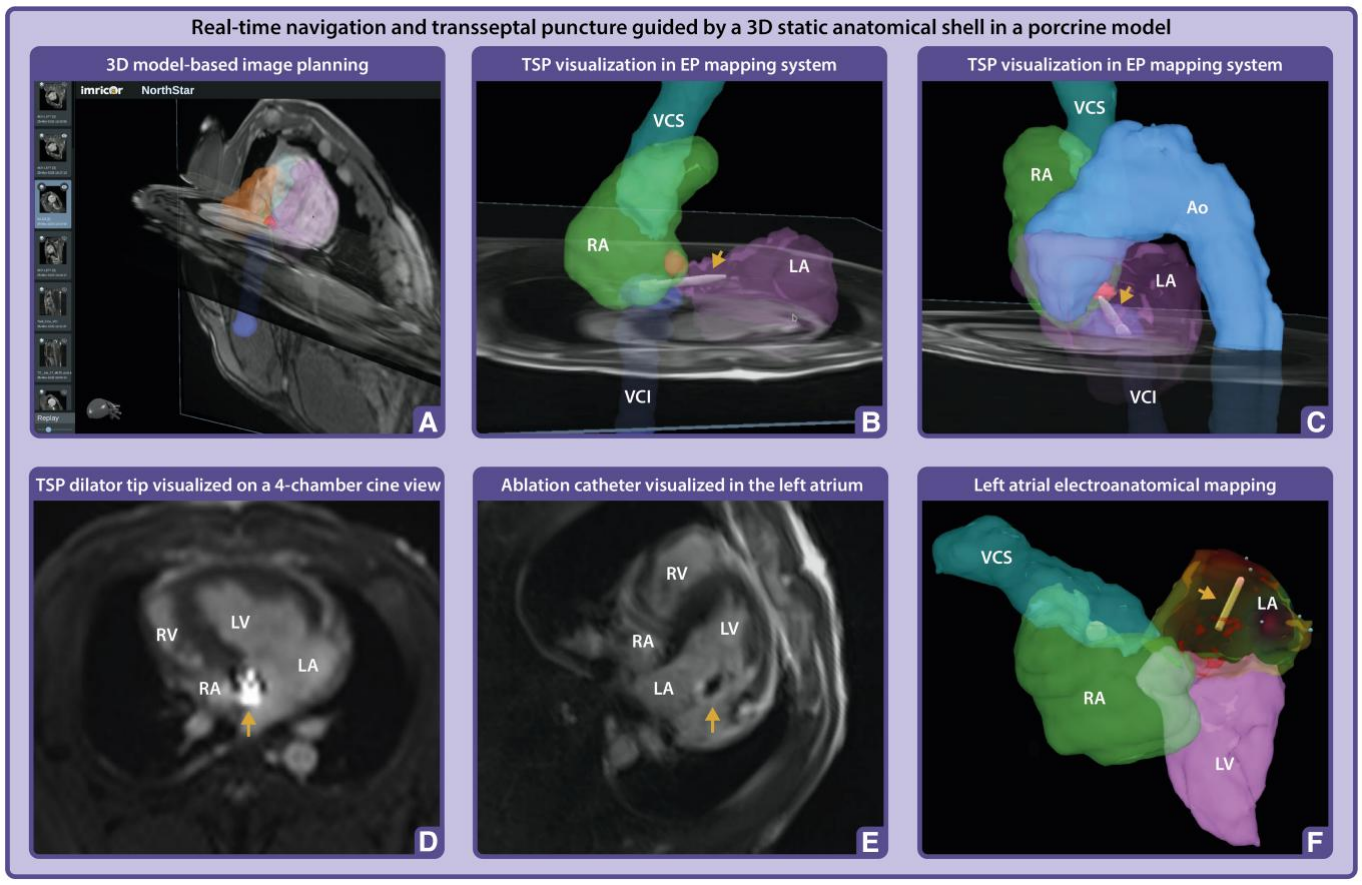
Real-time navigation and TSP guided by a 3D static anatomical shell in a porcine model. (*A*) Navigation of the MR-compatible TSP set within a 3D static anatomical shell imported into the NorthStar Mapping System, supplemented with adjustable 2D image planes to provide additional spatial orientation and visualization of cardiac motion. (*B–C*) The TSP set was navigated to the foramen ovale (highlighted in red segmentation), with positioning verified across multiple image planes to ensure accurate alignment for septal puncture. The dilator tip is projected onto the shell (arrow). (*D*) Positioning of the dilator tip confirmed on a real-time 4-chamber cine image using active catheter imaging, where the tip appears as a bright signal focus, caused by the built-in micro coil (arrow). (*E*) Following TSP and advancement of the ablation catheter into the left atrium, a 4-chamber cine image confirmed its position, visualized by the catheter tip artefact (arrow) in the left atrium. (*F*) The ablation catheter (arrow) projected in the Mapping System during activation mapping of the left atrium. Ao, Aorta; LA, left atrium; LV, left ventricle; RA, right atrium; RV, right ventricle; VCI, inferior vena cava; VCS, superior vena cava.

A vendor-neutral MR-compatible 3D navigation and electro-anatomical mapping (EAM) system (NorthStar, Imricor Medical Systems, Burnsville, MN, USA) can complement conventional 2D visualization of the dilator by enabling real-time 3D tracking of its position. This is achieved through miniature built-in receive coils within the tip of the dilator, which interface with the MRI scanner to localize its position. To support this functionality, a full-volume anatomical dataset of the heart and surrounding structures must first be acquired using an ECG-triggered, respiratory-navigated 3D steady-state free precession sequence. From this dataset, a 3D shell of the cardiovascular anatomy can be generated via segmentation, for example using ADAS 3D software (ADAS 3D Medical, Barcelona, Spain). Specific landmarks, such as the fossa ovalis area, can be marked to indicate the target, and the shell with landmarks can then be exported and uploaded to the navigation and mapping system (*Figure 3*, Panel A and B).

### Real-time integration of 2D cine and 3D anatomical roadmaps

The NorthStar platform integrates the 2D real-time cine imaging with the segmented static 3D anatomical shell to account for respiratory and cardiac motion during TSP. Moreover, the navigation system interfaces with the MRI scanner and can remotely execute validated scan protocols, including those designed for MR-conditional device tracking.^5^ This combined approach provides complementary spatial and temporal information: the 3D shell serves as a static anatomical roadmap for target localization, while real-time cine imaging ensures continuous visualization of catheter movement within the beating heart. This integration enhances precision in navigating the dilator toward the fossa ovalis and enables responsive adjustment to physiological motion. Unlike conventional EAM systems, which require registration or fusion with pre-acquired images, MR-guided navigation provides real-time alignment of anatomy and instrumentation, potentially improving spatial accuracy, procedural confidence, and safety during TSP.

## Real-time MR-guided TSP in a porcine model

To translate this approach into practice, we performed a real-time MR-guided TSP in a porcine model. The experimental protocol was reviewed and approved by the Institutional Animal Care and Use Committee at BioSim, LLC, Brooklyn Park, MN, US. Animal welfare and adherence to the protocol was overseen by BioSim, LLC representatives during the procedure performed at Imricor Medical Systems iMRI in Burnsville, MN, US.

As described above, an MR angiogram was required to enable detailed segmentation of the heart. The segmented 3D whole heart shell was subsequently imported into the NorthStar Mapping System. Subsequently, an MR-compatible sheath (Imricor Medical, Burnsville, MN, USA) was advanced via the femoral vein into the RA. The entire procedure was tracked in NorthStar, where the tip of the dilator was visualized within the static 3D shell of the heart (*Figure 3*, Panel B and C). Using the MR-compatible needle, the TSP was successfully performed under real-time MR-guidance, and the ablation catheter was navigated into the left atrium (LA). On real-time cine imaging, the catheter tip artefact was clearly visible within the LA cavity (*Figure 3*, Panel D and E). Local LA activation times were subsequently recorded to create a LA activation map (*Figure 3*, Panel F). Of note, this MR-compatible TSP set is one of the investigational products in the recently initiated multicenter, multinational VISABL-VT trial (NCT05543798), where it can be used to enable LA access for MR-guided left-sided ventricular tachycardia ablation.

## Challenges

Despite these advances, several important challenges remain. At present, only a single investigational MR-compatible TSP set is available for use under real-time MR-guidance. This system lacks CE and FDA certification and has not yet been evaluated in human subjects, underscoring the preliminary nature of its development. The necessary certification steps will need to be achieved through dedicated studies, such as the recently initiated VISABL-VT trial. Furthermore, the temporal resolution of real-time MRI remains inferior to that of conventional fluoroscopy, which may compromise the accurate visualization of catheter movement, positioning, and tenting dynamics during septal engagement, despite unparalleled image quality. Although the investigational system incorporates micro-coils within the dilator to enable active tracking, the accompanying sheath is devoid of such tracking capabilities. This limits comprehensive visualization and spatial orientation within MRI-based mapping environments. Finally, the development of MR-compatible guidewires that integrate optimal handling characteristics with reliable visibility through passive markers in both 2D and 3D MR environments remains a major technical challenge. The performance of the current generation of MR-compatible guidewires is still suboptimal, limiting procedural efficiency and thereby hindering widespread clinical adoption.

## Conclusion

The feasibility of MR-guided TSP using iCMR continues to gain momentum as innovations in MR-compatible instruments and procedural techniques advance. The successful adaptation of an MR-compatible TSP set, combined with sophisticated imaging guidance from real-time MR and 3D visualization systems, demonstrates the growing potential for MRI to guide complex left heart cardiovascular interventions. Nonetheless, further improvements in device visibility and regulatory approval, combined with faster real-time MRI imaging, are essential for translation into clinical practice.

## Data Availability

No new data were generated or analysed in support of this research.

## Lead author biography

Luuk Hopman completed his PhD at the Department of Cardiology, Amsterdam UMC, in 2024, focusing on cardiac MRI to investigate atrial myopathy in atrial fibrillation patients. He continued his research as a postdoctoral research fellow in cardiac MRI and clinical electrophysiology at Amsterdam UMC. His work includes characterizing atrial remodelling post-ablation, visualizing ablation lesions, evaluating responses to various pacing strategies using cardiac MRI, and using cardiac MRI for risk stratification in ICD patients. In recent years, he has also played an active role in interventional MRI, contributing to real-time MRI-guided interventions such as ablation procedures within the MRI scanner.
